# Association of *FCGR2A* rs1801274 and *FCGR3A* rs396991 polymorphisms with various autoimmune diseases: a meta-analysis

**DOI:** 10.3389/fimmu.2025.1661502

**Published:** 2025-09-10

**Authors:** Elena Thaler, Maike Bublitz, Martin Wipplinger, Christoph Gassner, Hanno Ulmer

**Affiliations:** ^1^ Institute of Translational Medicine, Faculty of Medical Sciences, Private University in the Principality of Liechtenstein (UFL), Triesen, Liechtenstein; ^2^ Institute of Clinical Epidemiology, Public Health, Health Economics, Medical Statistics and Informatics, Medical University of Innsbruck, Innsbruck, Austria

**Keywords:** FCGR2A, FCGR3A, single nucleotide polymorphism, genetic variants, autoimmune diseases, meta-analysis, genetic association, Fc gamma receptor

## Abstract

**Objectives:**

The aim of this systematic review with meta-analysis was to examine the association between the polymorphisms rs1801274 (*FCGR2A *H131R) and rs396991 (*FCGR3A* F158V) and susceptibility to autoimmune diseases (ADs), with a focus on the progress and novelty of studies published over the last two decades.

**Methods:**

A meta-analysis systematically evaluated *FCGR2A/3A* gene variants in autoimmune diseases (ADs) using four genetic models: dominant, recessive, overdominant, and allelic contrast.

**Results:**

The *FCGR3A* F158V polymorphism was significantly associated with immune thrombocytopenia in all four genetic models tested (dominant: OR = 2.67, 95% CI 1.94-3.67, for FV + VV vs. FF, recessive: OR = 2.38, 95% CI 1.78-3.19, for VV vs. FF + FV, overdominant: OR = 1.58, 95% CI 1.15-2.17, for FV vs. FF+VV, and allele comparison: OR = 1.97, 95% CI 1.70-2.29, for V vs. F, in the overall analyses). Statistically significant associations were also found between rheumatoid arthritis and *FCGR3A* F158V polymorphisms (recessive: OR = 1.36, 95% CI 1.09-1.69, for VV vs. FF + FV, and allele comparison: OR = 1.15, 95% CI 1.03-1.29, for V vs. F, in the overall analyses). Conversely, the overall analysis identified a negative association between the *FCGR2A* H131R polymorphism and rheumatoid arthritis in two genetic models (dominant: OR 0.83, 95% CI 0.69-1.00, for HR + RR vs. HH; allelic comparison: OR 0.86, 95% CI 0.76-0.97, for R vs. H).

**Conclusion:**

This meta-analysis revealed an association between *FCGR3A* V158 and an increased risk of immune thrombocytopenia and rheumatoid arthritis. However, this polymorphism is likely to explain only part of the pathogenesis of both diseases. Conversely, a protective association was found between *FCGR2A* R131 and rheumatoid arthritis. Nevertheless, the quantification of the total genetic contribution of a single gene remains challenging.

## 1 Introduction

Autoimmune diseases (ADs) are characterized by intricate and multifactorial pathophysiological processes involving dysregulation of both innate and adaptive immune responses. The etiology and progression of ADs are influenced by a complex interplay of genetic predisposition and environmental triggers. Epidemiological studies estimate that the global prevalence and incidence of ADs affect approximately 7.6–9.4% of the population (1), posing substantial challenges for healthcare systems worldwide. Although advances in therapeutic interventions have significantly improved the outcomes and quality of life for patients, many of the molecular and immunological pathways underlying these disorders remain incompletely understood, necessitating continued research to approach the early on diagnosis and development of targeted therapies.

Recent advances in genetic research have begun to shed light on some of the molecular mechanisms underlying autoimmunity. Polymorphisms in genes such as HLA, CTLA4, and IL2RA are now recognized as major contributors to the onset and progression of various autoimmune diseases (2–4). Additionally, variants in highly polymorphic Fc gamma receptor (FcγR) genes, which mediate IgG antibody recognition, have been linked to several ADs (5). In particular, the H131R variant in *FCGR2A* (rs1801274) and the F158V variant in *FCGR3A* (rs396991) are among the most extensively studied single nucleotide variants (SNVs) in this context.

**Figure 1:**
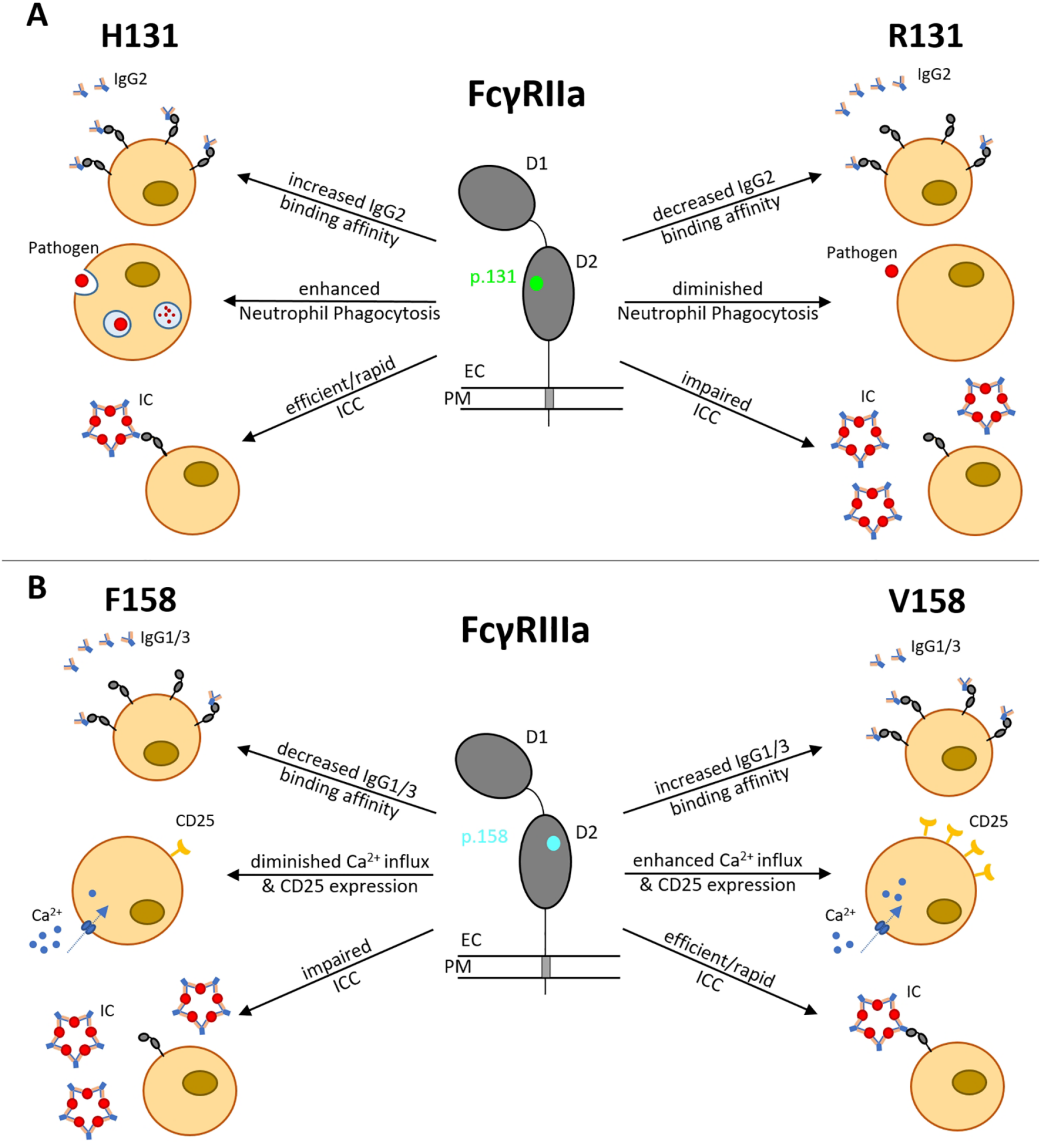
Functional consequences of FCGR2A H131R and FCGR3A F158V polymorphisms. **(A)** H131R (green) is located in the IgG-binding domain of FcγRIIa, influencing the binding affinity to IgG2, phagocytosis activity in neutrophils and ICC. **(B)** F158V (blue) is located in the IgG-binding domain of FcγRIIIa, influencing the binding affinity to IgG1/3, CD25 expression rate, Ca^2+^ influx and ICC. D1, Ig-like domain 1; D2, Ig-like domain 2; EC, extracellular; PM, plasma membrane; IC, immune complex; ICC, immune complex clearance.

The *FCGR2A* gene is located on chromosome 1q23 and comprises 7 exons spanning ~15.58 kb (6). Its protein product, Fcγ receptor IIa (FcγRIIa), acts as a low-affinity receptor for monomeric IgG, but also forms interactions with larger immune complexes (7). FcγRIIa (CD32a) is an integral membrane protein with two extracellular Ig-like domains and a cytoplasmic tail containing an immunoreceptor tyrosine-based activation motif (ITAM) (8). This receptor is expressed by most leucocytes, including monocytes, dendritic cells, macrophages, natural killer cells, platelets and endothelial cells, and a subpopulation of T-cells. The H131R polymorphism substitutes histidine (H) with arginine (R) at position 131 within the second Ig-like domain of the FcγRIIa receptor, altering its ability to bind IgG2 antibodies (9) (**Figure 1**). While the H131 (‘wild-type’) allele enhances immune defense, it can also promote inflammation and tissue damage, contributing to autoimmune diseases such as rheumatoid arthritis (RA), Graves’ disease, ulcerative colitis, childhood immune thrombocytopenia (ITP), and Kawasaki disease (7). However, the R131 (‘risk’) allele’s reduced immune activation is linked to a higher risk of infections like sepsis (10) and is also associated with systemic lupus erythematosus (SLE) (11) due to less efficient clearance of immune complexes (**Figure 1**). Hence, both H and R alleles and their resulting phenotypes demonstrate the delicate effect of this FcγRIIa R/H131 polymorphism in regulating the immune responses, with each genotype predisposing individuals to different risks of inflammatory or infectious diseases.

The *FCGR3A* gene is located on chromosome 1q23 and contains 7 exons spanning ~ 8.3 kb (12). It encodes the low-affinity Fcγ receptor IIIa (FcγRIIIa), which binds immunoglobulin G (IgG)-containing immune complexes (ICs) to mediate IC clearance (13), antibody-dependent cellular cytotoxicity (ADCC) and inflammatory cytokine release. The *FCGR3A* F158V polymorphism substitutes phenylalanine (F) with valine (V) at position 158 of FcγRIIIa (CD16a), also in the second IG-like domain, increasing receptor affinity for IgG1 and IgG3, and enhancing antibody-dependent cellular cytotoxicity (ADCC) by strengthening interactions with natural killer cells (NK) and macrophages, leading to hyperactive immune responses (14). The F158 (‘wild-type’, low-affinity) allele of the *FCGR3A* F158V polymorphism has a lower binding affinity of FcγRIIIa for IgG1 and IgG3. This leads to impaired immune complex (IC) clearance and dysregulated B - cell activation (15) (**Figure 1**). These pleiotropic effects therefore link the F158 variant to autoimmune pathogenesis, infectious disease susceptibility and variability in therapeutic outcomes (14), as well as reducing self-tolerance and predisposing individuals to SLE and lupus nephritis (LN) (7).

Higher-affinity (‘risk’) variant V158 enhances FcγRIIIa binding to IgG1 and IgG3, thereby improving IC clearance, but also increasing the risk of pathological immune overactivation.

Previous studies have either reported a single polymorphism across multiple autoimmune diseases (6) or several polymorphisms within a single disease (16–20), but comprehensive analyses examining multiple polymorphisms across multiple diseases are limited. Therefore, our systematic review with meta-analysis assesses and reports a structured, transparent summary of the current knowledge of *FCGR2A* (rs1801274) and *FCGR3A* (rs396991) polymorphisms in autoimmune diseases [immune thrombocytopenia (ITP), systemic lupus erythematosus (SLE), rheumatoid arthritis (RA), Guillain-Barré syndrome (GBS), celiac disease (CD)]. We hypothesized that these functional FcγR gene variants influence disease susceptibility, with risk associations varying by ethnicity, disease subtype and age (in ITP).

## 2 Materials and methods

### 2.1 Literature search and inclusion criteria

A comprehensive literature search was performed to examine the association between *FCGR2A* rs1801274 and *FCGR3A* rs396991 polymorphisms and autoimmune diseases. The following terms were searched: ″Fcγ receptor″, ″Fc gamma receptor″, ″FCGR″, ″CD32″, ″CD16″, ″polymorphism″, ″variant″, ″mutation″, ″autoimmune disease″ in four source databases: PubMed ( *https://pubmed.ncbi.nlm.nih.gov/* *)*, Google Scholar ( *https://scholar.google.com/* ), Cochrane Library ( *https://www.cochranelibrary.com/* *)* and Science.gov ( *https://www.science.gov/* *)* between January 1^st^, 2004 and October 14^th^, 2024, and relevant articles were identified for further filtering.

In line with the research objective and the planned meta-analysis, the following criteria had to be met by the included studies: (a) case-control studies investigating *FCGR* polymorphism (*FCGR2A* H131R or *FCGR3A* F158V) in relation to ITP, SLE, RA, GBS or CD; (b) providing data for the calculation of odds ratios (ORs) and 95% confidence intervals (CIs); (c) the full text had to be available in English. Studies were excluded if they met any of the following criteria: (a) not related to *FCGR2A/3A* polymorphisms and autoimmune disease; (b) animal or cancer studies; (c) no control group included; (d) case reports or case series.

### 2.2 Data extraction and quality assessment

The following information was extracted from the eligible studies: (a) the first author’s name; (b) the year of publication; (c) the country and ethnicity of the participants; (d) the sample size; and (e) the genotypic distributions of *FCGR2A/3A* polymorphisms in cases and controls.

Hardy-Weinberg equilibrium (HWE) was assessed for each study using Chi-squared tests to determine whether observed genotype frequencies deviated significantly from expected frequencies. HWE *P* values were calculated using Meta Genyo (21), with *P* values ≤ 0.05 indicating statistically significant departure from equilibrium assumptions.

**Figure 2:**
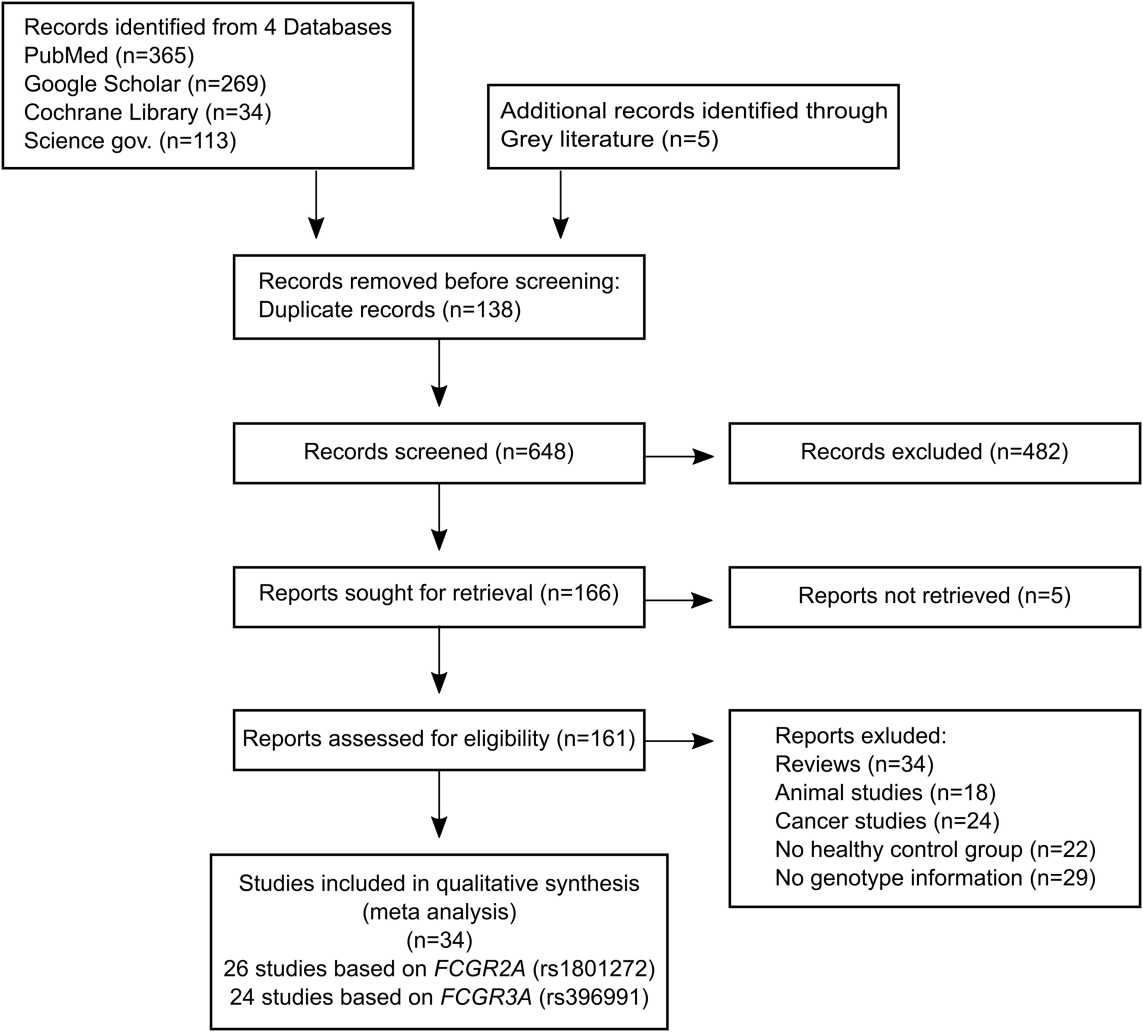
Flowchart illustrating the study selection process.

The Newcastle-Ottawa scale (NOS) was used to evaluate the quality of the eligible studies (22). The NOS has a score range of zero to nine, and studies achieving a score of more than seven were regarded as high-quality data. A flowchart illustrating the study selection process is given in **Figure 2**.

### 2.3 Statistical analyses

Statistical meta-analyses were performed using RStudio 4.4.3 with the ‘metafor’ package (version 4.8-0), supplemented by Meta Genyo (21) (https://metagenyo.genyo.es), and MetaAnalysisOnline (23) (https://metaanalysisonline.com) platforms. The association between *FCGR2A* rs1801274 and *FCGR3A* rs396991 polymorphisms and the onset of autoimmune diseases was evaluated using odds ratios (ORs) — a measure of association strength — and 95% confidence intervals (CIs), which reflect the precision of the estimates. Four genetic models were evaluated, including dominant (variant carrier vs. wild-type homozygote), recessive (variant homozygote vs. others), overdominant (heterozygote vs. combined homozygotes), and allelic (variant allele vs. wild-type allele) models.

The statistical significance of pooled ORs was determined using Z tests, with *P* values ≤ 0.05 considered statistically significant. Heterogeneity between studies was quantified using the I² statistic, where I² values of 25%, 50%, and 75% indicated low, moderate, and high heterogeneity, respectively (24). Given the expected clinical and methodological diversity across studies, random-effects models (DerSimonian and Laird method (25)) were employed for all analyses.

**Figure 3:**
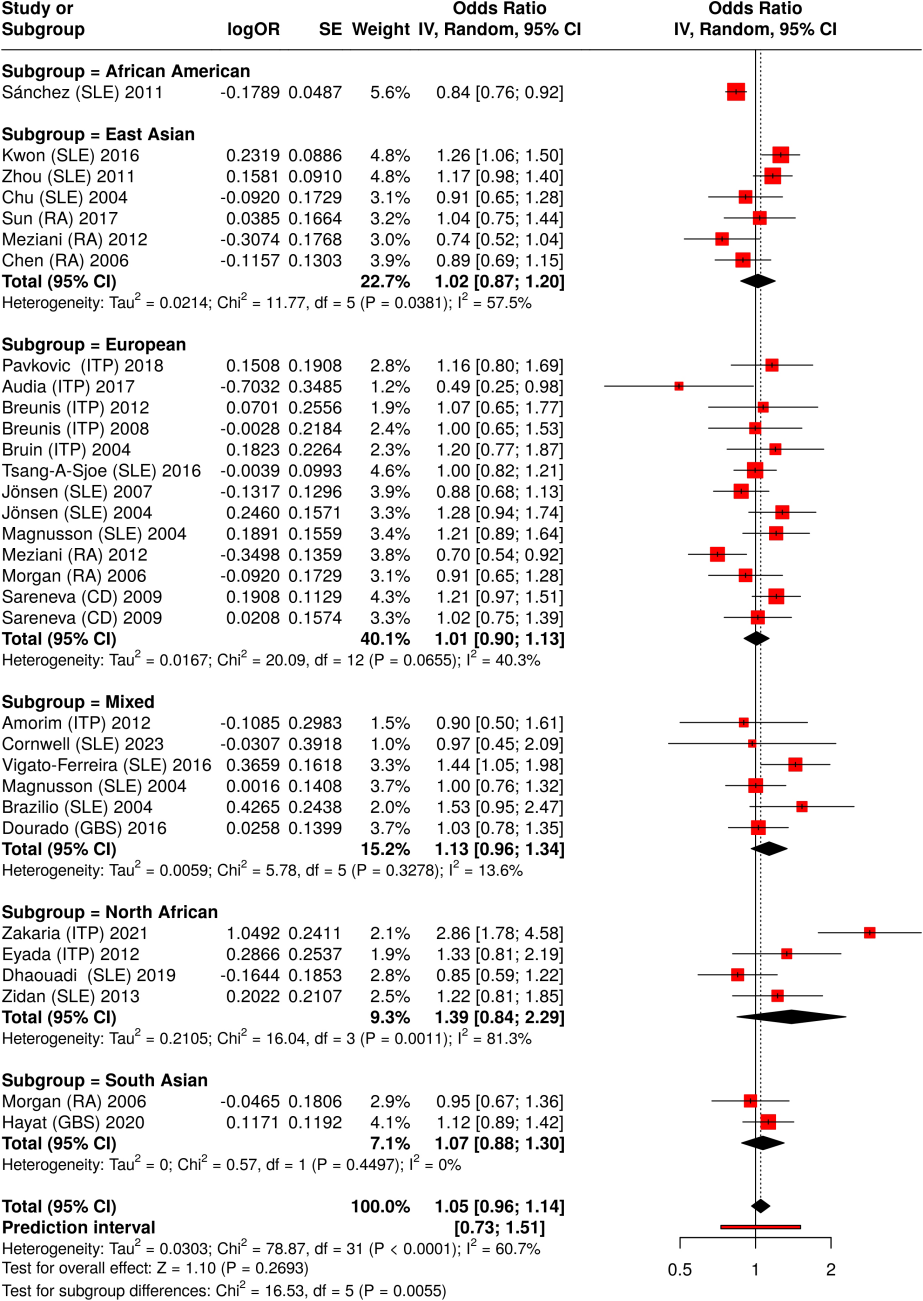
Meta-analysis of all pooled studies of *FCGR2A* rs1801274 polymorphisms by subset and summarized across all ADs.

**Figure 4:**
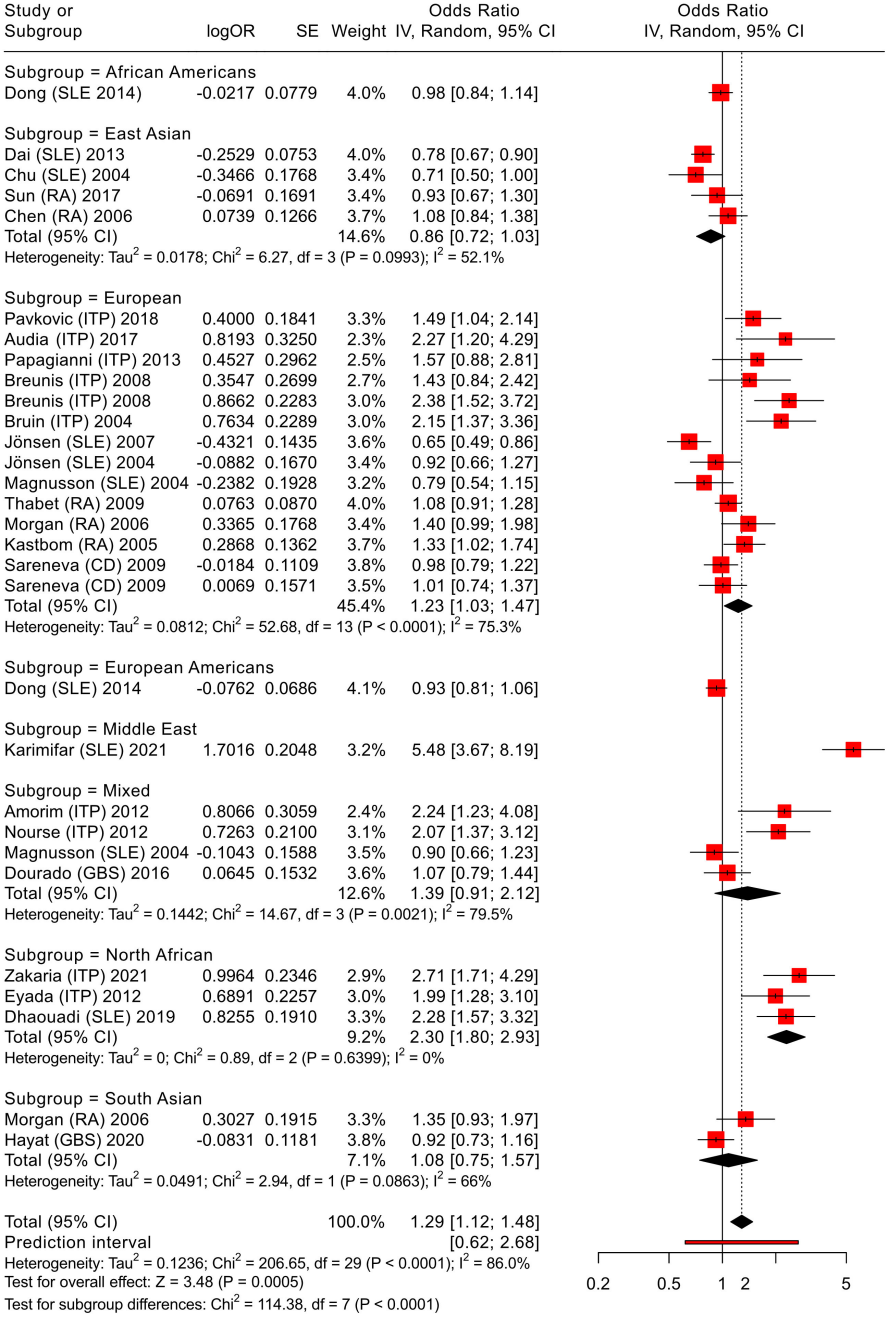
Meta-analysis of all pooled studies of *FCGR3A* rs396991 polymorphisms by subset and summarized across all ADs.

To explore potential sources of heterogeneity, subgroup analyses were conducted. The primary analyses were based on ethnicity (European, East Asian and North African populations) for each polymorphism in a specific disease. In the second analysis, several other populations were included in the studies’ pooled meta-analysis, which grouped each polymorphism with all diseases, as shown in **Figures 3** and **4**. For immune thrombocytopenia (ITP) studies, additional stratification by age (pediatric vs. adult) was performed in the primary analysis.

Sensitivity analyses were carried out by sequentially excluding individual studies to assess their impact on the overall effect magnitude.

Results were visualized using forest plots displaying individual and pooled effect sizes with corresponding 95% CIs. These forest plots illustrate the strength and direction of associations across individual studies, as well as the overall pooled population estimates. Publication bias was evaluated using Egger’s regression test (26). All statistical analyses were conducted in accordance with the Preferred Reporting Items for Systematic Reviews and Meta-Analyses (PRISMA) guidelines (27).

## 3 Results

### 3.1 Study selection

Our research strategy identified 781 potentially relevant articles from the four different databases PubMed, Google Scholar, Cochrane Library and Science.gov. A total of 648 records were retrieved after removing duplicates. After excluding irrelevant articles, 161 articles were retrieved for further evaluation. Upon reading the full text, 127 articles were subsequently excluded and a further 34 eligible studies (ITP - 9 studies (28–36), SLE - 16 studies (37–52), RA - 6 studies (53–58) Guillain-Barré syndrome (GBS) - 2 studies (56, 60) or celiac disease (CD) - 1 study (61)) were included for further quantitative analysis (**Figure 2**). All selected studies are given in **Table 1**.

**Table 1:** The characteristics of included studies for *FCGR* polymorphism and various autoimmune diseases.

First author (y)	Country	Ethnicity	Type of disease	Sample size^†^	Cases	Controls	*P* value for HWE	NOS score
*FCGR2A* H131R					HH/HR/RR			
Zakaria (2021)* (28)	Egypt	North African	Childhood-onset ITP	80/80	18/46/16	56/8/16	0.000	7
Pavkovic (2018) (29)	Macedonia	European	Adult-onset ITP	125/120	50/58/17	55/50/15	0.494	8
Audia (2017) (30)	France	European	Adult-onset ITP	24/108	12/10/2	32/54/22	0.928	7
Amorim (2012) (33)	Brazil	Mixed	Childhood-onset ITP	33/73	10/18/5	20/40/13	0.365	8
Eyada (2012)** (32)	Egypt	North African	Childhood-onset ITP	92/90	65/8/19	72/18/0	0.292	8
Breunis (2008) (35)	Netherlands	European	Adult-onset ITP	44/100	10/26/8	28/52/20	0.641	8
Breunis (2008) (35)	Netherlands	European	Childhood-onset ITP	72/100	25/28/19	28/52/20	0.641	8
Bruin (2004) (36)	Netherlands	European	Childhood-onset ITP	52/154	12/26/14	40/82/32	0.400	8
*FCGR3A* F158V					FF/FV/VV			
Zakaria (2021) (28)	Egypt	North African	Childhood-onset ITP	80/80	8/58/14	40/32/8	0.670	7
Pavkovic (2018) (29)	Macedonia	European	Adult-onset ITP	125/120	40/52/33	52/46/22	0.046	8
Audia (2017) (30)	France	European	Adult-onset ITP	24/108	6/12/6	52/46/10	0.894	7
Papagianni (2013) (31)	Greece	European	Childhood-onset ITP	53/45	6/46/1	15/29/1	0.004	8
Amorim (2012) (33)	Brazil	Mixed	Childhood-onset ITP	32/73	10/10/12	36/25/12	0.047	8
Nourse (2012) (34)	Australia	Mixed	Adult-onset ITP	100/100	27/52/21	48/44/8	0.634	8
Eyada (2012) (32)	Egypt	North African	Childhood-onset ITP	92/89	24/58/10	47/36/6	0.800	8
Breunis (2008) (35)	Netherlands	European	Adult-onset ITP	44/98	19/17/8	48/42/8	0.778	8
Breunis (2008) (35)	Netherlands	European	Childhood-onset ITP	72/98	16/40/16	48/42/8	0.778	8
Bruin (2004) (36)	Netherlands	European	Childhood-onset ITP	53/154	12/27/14	66/73/15	0.421	8
*FCGR2A* H131R					HH/HR/RR			
Cornwell (2021) (37)	USA	Mixed	SLE	51/18	12/22/17	4/8/6	0.637	8
Dhaouadi (2019) (38)	Tunisia	North African	SLE	137/100	41/50/46	24/40/36	0.060	7
Tsang (2016) (39)	Netherlands	European	SLE	266/919	78/134/54	269/463/187	0.634	7
Vigato-Ferreira (2016) (40)	Brazil	Mixed	SLE	157/160	23/75/59	35/82/43	0.727	7
Kwon (2016) (41)	Korea	East Asian	SLE	656/622	339/260/57	359/227/36	0.988	8
Zidan (2013) (42)	Egypt	North African	SLE	90/90	20/45/25	22/50/18	0.282	8
Zhou (2011) (43)	China	East Asian	SLE	589/477	238/269/82	209/220/48	0.370	7
Sánchez (2011) (44)	USA	African-American	SLE	1512/1788	490/741/281	491/892/405	0.997	8
Jönsen (2007) (45)	Sweden	European	SLE	323/200	105/158/60	58/99/43	0.950	8
Jönsen (2004) (46)	Sweden	European	SLE	143/200	27/70/46	49/100/51	0.999	8
Magnusson (2004) (47)	Sweden	European	SLE	136/224	26/67/43	48/121/55	0.223	8
Magnusson (2004) (47)		Mixed	SLE	189/224	43/97/49	48/121/55	0.223	8
Brazilio (2004) (48)	Brazil	Mixed	SLE	119/48	29/43/47	13/25/10	0.751	7
Chu (2004) (49)	China	East Asian	SLE	163/129	72/70/21	53/58/18	0.739	8
*FCGR3A* F158V					FF/FV/VV			
Karimifar (2021) (50)	Iran	Middle Eastern	SLE	143/95	25/17/101	42/35/18	0.038	7
Dhaouadi (2019) (38)	Tunisia	North African	SLE	137/100	28/64/45	43/42/15	0.376	7
Dong (2014) (51)	USA	European Americans	SLE	834/1185	392/370/72	517/564/104	0.004	8
Dong (2014) (51)		African Americans	SLE	648/953	289/283/76	413/431/109	0.829	8
Dai (2013) (52)	China	East Asian	SLE	732/886	376/308/48	381/427/78	0.006	7
Jönsen (2007) (45)	Sweden	European	SLE	323/200	200/108/15	99/84/17	0.891	8
Jönsen (2004) (46)	Sweden	European	SLE	143/200	68/61/14	90/88/22	0.944	8
Magnusson (2004) (47)	Sweden	European	SLE	103/221	56/43/4	109/94/18	0.717	8
Magnusson (2004) (47)		Mixed	SLE	178/221	96/67/15	109/94/18	0.717	8
Chu (2004) (49)	China	East Asian	SLE	163/129	76/74/13	48/63/18	0.711	8
*FCGR2A* H131R					HH/HR/RR			
Sun (2017) (53)	China	East Asian	RA	158/165	65/80/13	72/78/15	0.345	8
Meziani (2012) (54)	Japan	East Asian	RA	238/184	162/69/7	111/64/9	0.954	8
Meziani (2012) (54)		European	RA	182/273	63/88/31	68/137/68	0.952	8
Chen (2006) (55)	Taiwan	East Asian	RA	212/371	90/105/17	153/174/44	0.689	8
Morgan (2006) (56)	UK	Europe	RA	146/126	34/72/40	28/59/39	0.527	8
Morgan (2006) (56)		South Asian	RA	122/128	44/48/30	37/66/25	0.648	
*FCGR3A* F158V					FF/FV/VV			
Sun (2017) (53)	China	East Asian	RA	158/165	78/66/14	76/75/14	0.452	8
Thabet (2009) (57)	Netherlands	European	RA	945/388	353/442/150	148/189/51	0.440	8
Chen (2006) (55)	Taiwan	East Asian	RA	212/371	88/91/33	155/170/46	0.954	8
Morgan (2006) (56)	UK	European	RA	150/141	59/69/22	68/61/12	0.746	8
Morgan (2006) (56)		South Asian	RA	126/129	48/66/12	63/57/9	0.417	8
Kastbom (2005) (58)	Sweden	European	RA	181/362	70/85/26	168/161/33	0.528	8
*FCGR2A* H131R					HH/HR/RR			
Hayat (2020) (59)	Bangladesh	South Asian	GBS	303/302	114/124/65	116/136/50	0.347	7
Dourado (2016) (60)	Brazil	Mixed	GBS	140/362	26/74/40	74/182/106	0.798	7
*FCGR3A* F158V					FF/FV/VV			
Hayat (2020) (59)	Bangladesh	South Asian	GBS	303/302	120/143/40	110/150/42	0.420	7
Dourado (2016) (60)	Brazil	Mixed	GBS	134/363	66/60/14	180/148/35	0.571	7
*FCGR2A* H131R					HH/HR/RR			
Sareneva (2009) (61)	Finland	European	CD	270/450	92/131/47	178/210/62	0.996	8
Sareneva (2009) (61)	Finland	European	CD	139/198	42/69/28	61/98/39	0.975	8
*FCGR3A* F158V					FF/FV/VV			
Sareneva (2009) (61)	Finland	European	CD	270/450	90/132/48	148/220/82	0.988	8
Sareneva (2009) (61)	Finland	European	CD	139/198	44/68/27	63/97/38	0.951	8

HWE, Hardy-Weinberg Equilibrium; ITP, Immune thrombocytopenia; SLE, systematic lupulus erythematosus; RA, rheumatoid arthritis, GBS, Guillain-Barré syndrome; CD, celiac disease; NOS, Newcastle-Ottawa Scale.

^†^All studies used blood samples as source of DNA, with the following exceptions: Amorim (33) used blood and bone marrow; Audia (30) used spleen tissue; Breunis (35), Tsang (39) and Kwon (41) did not specify the source of DNA.

^*^Zakaria et al. (28): the control group’s genotype is changed and is not the same as in the original study.

^**^Eyada et al. (32): the genotype number for the control group was taken from Li et al. (16), not from the original study.

### 3.2 Study characteristics

Using the Newcastle-Ottawa scale (NOS), the quality of included studies ranged in between 7 and 8 and is shown in **Table 1**.

### 3.3 Overall and subgroup analyses for *FCGR2A* and *FCGR3A*

#### 3.3.1 FCGR2A

The data from the chosen studies, based on diverse autoimmune cohorts (ITP, SLE, RA, GBS, CD), revealed no statistically significant association between *FCGR2A* H131R and disease susceptibility across all genetic models tested (dominant, recessive, overdominant, allelic contrast) (**Supplementary Tables S1**-**S4**). However, marginal trends emerged in the dominant and allelic models in RA (**Table 2**), suggesting potential allele-specific effects.

**Table 2:** Overall and subgroup analyses of the *FCGR2A* H131R, rs1801274 polymorphism in RA.

Population	Number of studies	Sample size (cases/control)	Comparison	Test of Association†		
				OR	95% CI	*P* value
Overall	6	1058/1251	Dominant	0.83	0.69-1.00	**0.05**
			Recessive	0.79	0.62-1.01	0.06
			Overdominant	0.95	0.79-1.14	0.55
			Allele comparison	0.86	0.76-0.97	**0.02**
European	2	328/399	Dominant	0.73	0.50-1.07	0.10
			Recessive	0.71	0.50-1.00	**0.05**
			Overdominant	0.99	0.74-1.33	0.96
			Allele comparison	0.78	0.61-1.01	0.06
East Asian	3	608/724	Dominant	0.91	0.71-1.17	0.48
			Recessive	0.71	0.46-1.09	0.12
			Overdominant	1.01	0.79-1.29	0.95
			Allele comparison	0.90	0.76-1.07	0.22

†The significant *P* value is bold in the table; it shows an association between *FCGR2A* rs1801274 polymorphism and RA. OR, odds ratio quantifies the strength of association between carrying the R allele (risk allele) versus the H allele (wild type) and a binary outcome (disease risk). CI, the confidence interval estimates the range of possible true association strengths between alleles.

The development of RA appears to be less likely in people with the *FCGR2A* R131 allele, according to the overall population analysis. According to the allelic model (R vs. H), the R131 allele has been found to be associated with a reduced risk of RA (OR 0.86, 95% CI 0.76-0.97, *P* = 0.02) (**Table 2**). Similarly, in the dominant model (HR + RR vs. HH), individuals homozygous for the H131 allele (HH) showed a higher risk compared to R131 allele carriers (OR 0.83, 95% CI 0.69-1.00, *P* = 0.05) (**Table 2**). Further analysis divided by population or disease category revealed no statistically significant associations, potentially due to insufficient statistical power or the presence of confounding factors (**Table 2**, **Supplementary Tables S1**-**S4**).

In the subgroup analyses, the East Asian group demonstrated a significant association in three genetic models (dominant, recessive, and allele comparison) between *FCGR2A* and SLE (see **Supplementary Table S2**). Conversely, the European and North African subgroups did not demonstrate any substantial associations between ADs and the *FCGR2A* H131R polymorphism (**Supplementary Table S2**).

#### 3.3.2 FCGR3A

The *FCGR3A* F158V (rs396991) polymorphism demonstrated a statistically significant association with ITP susceptibility in the overall population analysis, across all four genetic models tested: dominant (OR = 2.67, 95% CI 1.94–3.67, *P* < 0.001, FV + VV vs. FF), recessive (OR = 2.38, 95% CI 1.78–3.19, *P* < 0.001, VV vs. FF + FV), overdominant (OR = 1.58, 95% CI 1.15-2.17, *P* = 0.005, FV vs. FF + VV). Furthermore, homozygotes for the V158 allele (VV) showed an increased risk compared to carriers of the F158 allele, in allele comparison (OR = 1.97, 95% CI 1.70-2.29, *P* < 0.001, V vs. F) (**Table 3**, **Supplementary Table S5**).

**Table 3:** Overall and subgroup analyses of the *FCGR3A* F158V, rs396991 polymorphism in ITP.

Population	Number of studies	Sample size (cases/control)	Comparison	Test of Association†		
				OR	95% CI	*P* value
Overall	10	675/965	Dominant	2.67	1.94-3.67	**< 0.001**
			Recessive	2.38	1.78-3.19	**< 0.001**
			Overdominant	1.58	1.15-2.17	**0.005**
			Allele comparison	1.97	1.70-2.29	**< 0.001**
Childhood-onset ITP	6	382/539	Dominant	3.47	2.40-5.02	**< 0.001**
			Recessive	2.57	1.71-3.86	**< 0.001**
			Overdominant	1.97	1.24-3.12	**0.004**
			Allele comparison	2.19	1.79-2.66	**< 0.001**
Adult-onset ITP	4	413/486	Dominant	1.86	1.34-2.57	**< 0.001**
			Recessive	2.20	1.45-3.35	**< 0.001**
			Overdominant	1.17	0.86-1.60	0.325
			Allele comparison	1.72	1.37-2.16	**< 0.001**
European	6	371/623	Dominant	2.21	1.56-3.13	**< 0.001**
			Recessive	2.37	1.63-3.44	**< 0.001**
			Overdominant	1.33	0.97-1.83	0.080
			Allele comparison	1.81	1.49-2.20	**< 0.001**
North African	2	172/169	Dominant	5.12	1.85-14.19	**< 0.001**
			Recessive	1.81	0.90-3.64	**0.002**
			Overdominant	3.08	1.97-4.80	0.096
			Allele comparison	2.31	1.68-3.18	**< 0.001**

†The significant P value is bold in the table; it shows an association between *FCGR3A* rs396991 polymorphism and ITP. OR, odds ratio quantifies the strength of association between carrying the V allele (risk type) versus F allele (wild allele) and a binary outcome (disease risk). CI, the confidence interval estimates the range of possible true association strengths between alleles.

In the childhood-onset ITP subgroup, significant associations were also detected in all four genetic models, consistent with the overall analysis. In the European population subgroup, for adult-onset ITP we observed significant associations in all models except the overdominant model (**Table 3**, **Supplementary Table S5**).

We performed further comprehensive analyses of diverse autoimmune populations (ITP, SLE, RA, GBS and CD), identical to those performed for *FCGR2A* rs1801274 (A>G), as detailed in **Supplementary Tables S5**-**S8**.

A statistically significant association between the *FCGR3A* F158V polymorphism and RA was identified in both the recessive model (OR = 1.36, 95% CI 1.09-1.69, *P* = 0.01, VV vs. FF + FV) and allele comparison (OR = 1.15, 95% CI 1.03-1.29, *P* = 0.02, V vs. F) (**Table 4**, **Supplementary Table S7**). Subgroup analyses by European ancestry yielded consistent positive results for both genetic models, in line with the overall analysis. No significant associations were found for the East Asian subgroup.

**Table 4:** Overall and subgroup analyses of the *FCGR3A* rs396991 polymorphism in RA.

Population	Number of studies	Sample size (cases/control)	Comparison	Test of Association†		
				OR	95% CI	*P* value
Overall	6	1772/1556	Dominant	1.14	0.97-1.34	0.12
			Recessive	1.36	1.09-1.69	**0.01**
			Overdominant	0.99	0.86-1.15	0.92
			Allele comparison	1.15	1.03-1.29	**0.02**
European	3	1276/891	Dominant	1.19	0.96-1.48	0.12
			Recessive	1.41	1.08-1.85	**0.01**
			Overdominant	1.00	0.84-1.20	0.99
			Allele comparison	1.21	1.02-1.42	**0.03**
East Asian	2	370/536	Dominant	0.96	0.73-1.25	0.76
			Recessive	1.23	0.81-1.85	0.33
			Overdominant	0.88	0.67-1.15	0.35
			Allele comparison	1.02	0.84-1.25	0.82

†The significant P value is bold in the table; it shows an association between *FCGR3A* rs396991 polymorphism and RA. OR, odds ratio quantifies the strength of association between carrying the V allele (risk type) versus F allele (wild allele) the and a binary outcome (disease risk). CI, the confidence interval estimates the range of possible true association strengths between alleles.

Across all analyses, a consistent relationship between the *FCGR3A* rs396991 polymorphism and the occurrence of both ITP and RA was observed (**Tables 3**, **4**, **Supplementary Tables S5**, **S7**). This polymorphism appeared to afford protection against SLE in the European subgroup (**Supplementary Table S6**). The association between *FCGR3A* rs396991 and susceptibility to several autoimmune diseases was confirmed in an analysis of the European population (**Tables 3**, **4**, **Supplementary Tables S5**, **S8**), but further studies with greater statistical power are needed to confirm these results.

### 3.4 Secondary analysis

#### 3.4.1 FCGR2A

In the secondary analysis, we split all available studies on *FCGR2A* rs1801274 into subgroups based on their proband ethnicity. By aggregating data from multiple studies, the meta-analysis (**Figure 3**) provides a more robust estimate of the genetic effect of *FCGR2A* rs1801274 on autoimmune disease susceptibility.

The combined meta-analysis showed no overall significant association, but subgroup analyses identified significant associations between general autoimmune disease and the H131R polymorphism in North Africans (allelic comparisons, OR = 1.39, 95% CI 0.84-2.29, *P* < 0.01, R vs. H), and East Asians (allelic comparisons, OR = 1.02, 95% CI 0.87-1.20, *P* = 0.04, R vs. H) (**Figure 3**, **Supplementary Table S9**). Pooled odds ratios (ORs) from allelic comparisons were calculated using a random-effects meta-analysis model (25) for all diseases associated with *FCGR2A* (rs1801274).

#### 3.4.2 FCGR3A

We applied an analogous analytical strategy to that used for *FCGR2A* polymorphism, as illustrated in **Figure 4**, to synthesize evidence from all studies. By combining findings from different populations, the analysis reduces the impact of insufficient statistical power in any one investigation and allows for a broader perspective on the role of *FCGR3A* in autoimmune disease susceptibility. The meta-analysis framework thus provides a more nuanced understanding of how this genetic F158V variant may influence disease risk across diverse autoimmune diseases.

The aim was to determine whether this genetic variant is linked to overall susceptibility to autoimmune diseases, or whether it is notable in a specific subgroup. The results show a significant association of allelic comparisons (OR = 1.29, 95% CI 1.12-1.48, *P* < 0.01, V vs. F, see **Figure 4**, **Supplementary Table S10**), supporting our initial hypothesis and confirming the findings from the above primary meta-analysis of ITP and RA cases. The pooled odds ratio was calculated using a random-effects model (25) based on allelic comparisons for all diseases associated with the *FCGR3A* rs396991 polymorphism. Notably, this association was particularly evident in the European subgroup (OR = 1.23, 95% CI 1.03-1.47, *P* < 0.01, V vs. F).

### 3.5 Sensitivity analysis

The pooled results remained unaltered in all comparisons, which suggests that our findings are statistically stable. The results were confirmed with three independent software applications.

### 3.6 Publication bias

The potential for publication bias was evaluated by Egger’s linear regression test, and a *P* value ≤ 0.05 was considered indicative of statistical publication bias (26). We cannot exclude the possibility of publication bias affecting our pooled estimates (see also Discussion and Limitations sections).

## 4 Discussion

The symptoms of ADs are wide-ranging, which can make diagnosis difficult, particularly in the early stages. In addition to environmental influences, genetic predisposition also plays an important role in the development and progression of diseases. Advances in genetic analysis have helped to identify genetic contributions, the genes themselves, and their polymorphisms, such as the polymorphisms in Fcγ receptors (FcγRs) examined in this study, in particular *FCGR2A* (rs1801274) and *FCGR3A* (rs396991). These genetic variants crucially modulate the handling of immune complexes (ICs) and inflammatory responses, thereby influencing susceptibility to autoimmune and inflammatory diseases.

In this systematic review with meta-analysis, we have analyzed two single-nucleotide polymorphisms (SNPs), *FCGR2A* (rs1801274) and *FCGR3A* (rs396991), which have been associated with ITP, SLE and RA in the past two decades. Due to the limited number of available studies on GBS and CD, no association was observed with those diseases in any of the four genetic models tested.

### 4.1 FCGR2A

The *FCGR2A* rs1801274 polymorphism is due to an A-to-G nucleotide exchange at coding nucleotide c.500 (NM_001136219.3), that encodes either histidine (H) or arginine (R) at amino acid position p.131 in the FcγRIIa receptor protein (62). This single amino acid substitution significantly influences the receptor’s binding affinity for IC handling: the H131 variant binds IgG2 and IgG3 with much higher affinity than R131 due to optimized electrostatic interactions with the Fc region (48). The reduced IgG2 affinity in R131 carriers specifically impairs neutrophil phagocytosis of IgG2-opsonized targets, while responses to IgG1/IgG3/IgG4 remain intact (63). This diminished IgG2 binding compromises the clearance of IgG2 immune complexes, allowing their deposition in tissues such as renal glomeruli and dermal vasculature (64, 65), which in turn promotes inflammation through FcγRIIa-mediated platelet activation and upregulation of endothelial adhesion molecules. These mechanisms contribute to thrombotic complications and accelerate atherosclerosis, particularly in SLE (66).

In addition, the R131 variant’s reduced IgG2 binding capacity appears to suppress neutrophil activation and matrix metalloproteinase (MMPs) release (67) which may help explain its association with reduced progression in RA despite its pro-inflammatory implications. This protective linkage was confirmed in our meta-analysis between the R131 polymorphism and RA in the overall population, suggesting a disease-specific influence on autoimmune pathogenesis.

The H131 variant demonstrates heightened binding affinity for IgG2 immune complexes (ICs) (68), facilitating efficient phagocytic clearance by neutrophils and macrophages. This increased efficiency not only enhances phagocytosis, neutrophil activation (69, 70), and IL-1β secretion (71), but also supports a more robust inflammatory response to IgG2 ICs. This enhanced clearance can trigger complement cascades and the production of pro-inflammatory cytokines, such as TNF-α and IL-6 (72). It can also promote sustained immune activation, driving chronic inflammation and the generation of autoantibodies — key pathogenic features of SLE, RA and multiple sclerosis (MS) (73). When this immune activation persists, the incomplete clearance of immune complexes (ICs) can lead to complications: these complexes may then accumulate in tissues such as the renal glomeruli and synovium (74), where complement-mediated lysis and cytokine-driven pathways can further exacerbate tissue injury and inflammation (75).

The higher prevalence of the H131 allele in Asian populations (76) as observed in our East Asian subgroup analysis by ethnicity, correlates with increased susceptibility to SLE (43) (**Supplementary Table S2**).

Therefore, the functional impact of the H131R polymorphism is context-dependent, modulating both immune defense and the risk of autoimmune tissue injury (**Table 5**).

**Table 5:** Functional consequences of *FCGR2A* (rs1801274) variants.

Key Features	H131	R131
IgG2 Binding Affinity	Higher	Lower
Neutrophil Phagocytosis	Enhanced	Diminished
Immune Complex Clearance	Efficient and rapid	Impaired
Diseases linked to *FCGR2A* H131R	Decreased inflammation; Increased risk for SLE, RA, MS	Decreased risk for RA; Increased risk of atherosclerosis in SLE

### 4.2 FCGR3A

The F158V polymorphism arises from a T-to-G substitution at coding nucleotide c.526 (NM_000569.8), substituting phenylalanine (F158) with valine (V158) in the receptor’s extracellular domain (77). The V158 variant increases binding affinity for IgG1/IgG3 by ~5-fold compared to F158 (78), likely due to valine’s smaller side chain reducing steric hindrance and optimizing hydrophobic interactions with the Fc region. The high-affinity V158 variant enhances antibody-dependent cellular cytotoxicity (ADCC) by strengthening interactions between FcγRIIIa and the Fc region of IgG1/IgG3 (79, 80) which affects neutrophil activation and effector functions, including phagocytosis and reactive oxygen species production. Functionally, natural killer (NK) cells from individuals homozygous for FcγRIIIa 158V display increased calcium influx, elevated CD25 expression, and accelerated apoptosis relative to those from FcγRIIIa 158F homozygotes, reflecting a more robust activation profile. Concurrently, FcγRIIIa 158V enhances antibody-dependent cellular cytotoxicity (ADCC) in NK cells by stabilizing FcγRIIIa engagement with IgG1/IgG3-opsonized targets (81), driving perforin/granzyme polarization inducing apoptosis.

In SLE, the V158 variant’s enhanced IgG1/IgG3 binding is hypothesised to exacerbate neutrophil extracellular trap (NET) formation and renal IC deposition (82), accelerating lupus nephritis (LN) (83). Contradictorily, the same mechanism may improve clearance of apoptotic debris, reducing autoantigen persistence and dampening chronic inflammation. This duality underscores the context-dependent influence of FcγRIIIa polymorphisms. In clinical settings, the V158 variant’s heightened signalling capacity augments the efficacy of anti-CD20 therapies such as rituximab (84), as pronounced FcγRIIIa clustering on natural killer cells facilitates more effective B-cell depletion and enhances therapeutic outcomes (79, 80). Consequently, the V158 variant is associated with superior B-cell depletion and improved clinical responses to anti-CD20 treatment. **Table 6** summarizes the functional implications of the F158V polymorphism.

**Table 6:** Functional consequences of *FCGR3A* (rs396991) variants.

Key Features	F158	V158
IgG1/IgG3 Binding Affinity	Lower	Higher
Cellular Effect (NK Cell ADCC, Calcium Influx, CD25, Apoptosis)	Diminished	Enhanced
Immune Complex Clearance	Impaired	Efficient and rapid
Inflammatory/Clinical Consequence	Weak	Strong
Diseases linked to *FCGR3A* F158V	Decreased chronic inflammation, ADCC responses	Increased risk of apoptosis, lupus nephritis in SLE

### 4.3 FcγRIIa H131R and FcγRIIIa F158V: a putative synergistic interplay

**Figure 5:**
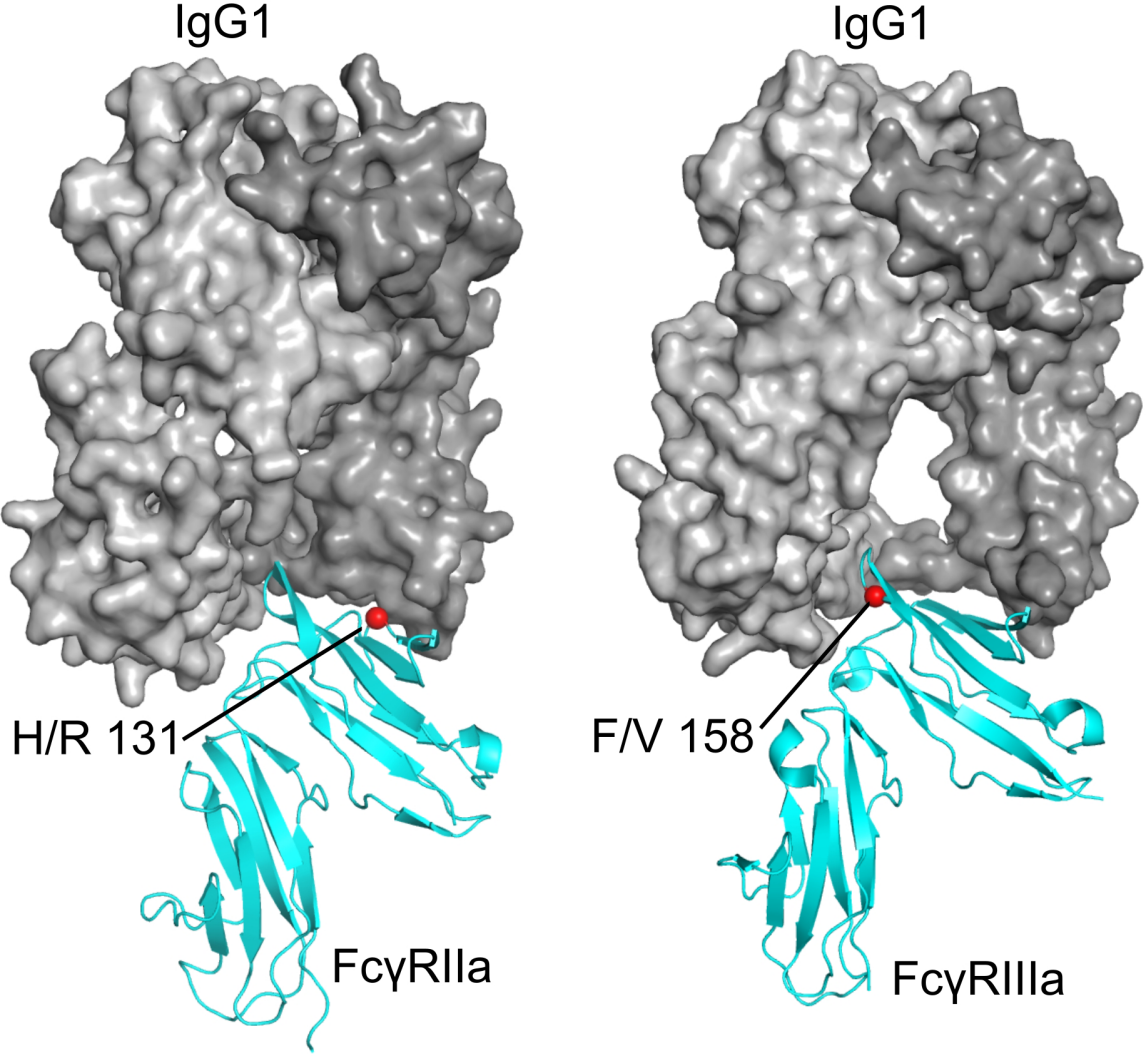
Positions of the FcγR polymorphisms investigated in this study. *Left*: FcγRIIa (cyan) bound to a human IgG1 Fc fragment (gray), with H/R 131 indicated by a red sphere (PDB ID 3RY4, 3RY6, PDB corresponding residue number 134) (87). *Right*: FcγRIIIa (cyan) bound to a human IgG1 Fc fragment (gray), with F/V 158 indicated by a red sphere (PDB ID 3SGJ) (88).

Accumulating evidence from our study and others highlights both the H131R and F158V polymorphisms as key modulators of immune regulation, although their direct mechanistic roles in autoimmune pathogenesis remains incompletely resolved (81, 85). As outlined in the report, both FcγRIIa and FcγRIIIa are characterized by co-dominantly expressed allelic variants that modulate their ligand-binding affinities, thereby shaping the magnitude and quality of cellular responses to ICs (85, 86). Structural data show that both polymorphisms are located directly within the binding interface between the receptor ectodomains and the IgG Fc regions (87, 88) (**Figure 5**).

The presence of functionally distinct polymorphisms in these receptors can synergistically alter effector cell activation, cytokine production, and the balance between IC clearance and inflammation. The combined inheritance of *FCGR2A* H131R and *FCGR3A* F158V polymorphisms may establish a “dual-hit” scenario, wherein compromised IC clearance due to FcγRIIa dysfunction is compounded by augmented FcγRIIIa-mediated inflammatory signaling. This interplay could amplify chronic immune activation, thereby accelerating tissue injury and promoting the progression of autoimmune diseases such as SLE and RA (**Table 7**).

**Table 7:** Functional consequences and pathogenic outcomes of *FCGR2A/3A* variants.

Polymorphism(s)	Functional Consequence	Pathogenic Outcome
*FCGR2A* R131	Impaired phagocytosis → Persistent immune complexes (ICs) → IC deposition in tissues	Tissue deposition, chronic inflammation
*FCGR3A* V158	Enhanced IgG1/IgG3 binding, increased ADCC Hyperresponsive effector cells (NK/macrophages)	Amplified tissue damage
Both (*2A* R131 + *3A* V158)	Synergetic effect: Persistent ICs + hyperresponsive effector cells create a feedback loop → Tissue-deposited ICs continuously activate V158-high-affinity receptors	Uncontrolled inflammation, accelerated end-organ damage

Reports on the pathogenic versus protective roles of these variants are conflicting. For instance, *FCGR3A* F158V has been linked to increased ITP and RA severity. In contrast, the *FCGR2A* H131R polymorphism has been identified as a risk factor for SLE. However, the present study only replicated this finding in the East Asian subgroup, and no clear effect on susceptibility for LN (89) was observed. The results suggest that the effect may vary between populations. The discrepancies highlight the complexity of Fcγ receptor biology and the influence of genetic background, environmental factors, and disease context.

Resolving these inconsistencies requires functional studies that elucidate how these polymorphisms collectively alter immune cell signalling networks, particularly within myeloid lineages such as neutrophils and macrophages. Future research should map the effects of *FCGR2A* and *FCGR3A* variants on IC handling, neutrophil activation, and B-cell tolerance. Such strategies, aligned with advances in genetics and immunology, will be essential to clarify these genetic associations and identify potential therapeutic targets in autoimmune disease.

### 4.4 Limitations

Several limitations of the present meta-analysis warrant consideration. The limited number of included studies for certain autoimmune diseases reduces the statistical power, increasing the risk of false-negative results. Variations in population characteristics, diagnostic criteria, reliability of the data, and genotyping methods may further limit comparability and applicability. Overall, heterogeneity was substantial, leading us to consistently apply random-effects models throughout our analyses. Random-effects models are the preferred method when heterogeneity is present between original studies, as they account for both within-study and between-study variance. Moreover, Egger’s linear regression test (*P* ≤ 0.05 threshold) identified publication bias across all comparisons, which may have influenced our outcome. Additionally, our analysis did not adjust for potential confounding variables such as sex, age, or environmental factors. These limitations indicate the requirement for future large-scale, well-controlled studies in diverse populations to confirm and extend our findings.

## 5 Conclusion

In summary, this meta-analysis found *FCGR3A* V158 to be associated with an increased susceptibility to two autoimmune diseases, namely immune thrombocytopenia (ITP) and rheumatoid arthritis (RA). However, the functional impact of the *FCGR3A* V158 polymorphism likely accounts for only a portion of the pathogenesis in both ITP and RA. Furthermore, our findings imply that the *FCGR2A* R131 allele may protect against RA, indicating a negative correlation with disease risk.

Additionally, the results suggest that both the *FCGR2A* (rs1801274) and *FCGR3A* (rs396991) polymorphisms may be associated with susceptibility to various autoimmune diseases in both European and East Asian populations. Future studies should use larger, well-designed case-control cohorts to improve statistical power and refine estimates of the effects of individual genes on the development of autoimmune diseases.

## Data Availability

Publicly available datasets were analyzed in this study. This data can be found here: PubMed (https://pubmed.ncbi.nlm.nih.gov/), Google Scholar (https://scholar.google.com/), Cochrane Library (https://www.cochranelibrary.com/), Science.gov (https://www.science.gov/).
