# Supplementary material for: Association of FCGR2A rs1801274 and FCGR3A rs396991 polymorphisms with various autoimmune diseases: a meta-analysis
Source: Front Immunol. 2025 Sep 10;16:1661502. doi: 10.3389/fimmu.2025.1661502 (PMC12457292; doi:10.3389/fimmu.2025.1661502)
Supplement: Supplementary file 1 [file DataSheet1.pdf]

Supplementary Material

Association of *FCGR2A* rs1801274 and *FCGR3A* rs396991 polymorphisms with various autoimmune diseases: A meta-analysis

Thaler E[1*], Bublitz M[1], Wipplinger M[1], Gassner C[1]†, Ulmer H[2]†

[1] Institute of Translational Medicine, Faculty of Medical Sciences, Private University in the Principality of Liechtenstein (UFL), 9495 Triesen, Liechtenstein.

[2] Institute of Clinical Epidemiology, Public Health, Health Economics, Medical Statistics and Informatics, Medical University of Innsbruck, Innsbruck, Austria.

[†]These authors share senior authorship

[*] **Correspondence:**
Corresponding Author
elena.thaler@ufl.li

1 Supplementary Tables 1-10

Supplementary Table 1: Meta Analysis of the *FCGR2A* rs1801274 in ITP

Disease	Population /ITP group	No. of studies	Sample size (cases/controls)	Comparison	Test of Association					Test of Heterogeneity			Publication bias
					OR	95% CI	Z	*P* Value	Model	Q	*P* Value	I^2 (%)	Egger's test *P* Value
ITP	Overall	8	522/825	Dominant	1.02	0.78-1.34	0.14	0.89	R	8.17	0.32	14.36	0.19
				Recessive	1.26	0.80-1.98	1.01	0.31	R	12.04	0.10	41.84	0.23
				Overdominant	0.81	0.61-1.07	-1.47	0.14	R	9.48	0.23	26.13	0.27
				Allele comparison	1.12	0.86-1.47	0.85	0.40	R	17.13	0.02	59.12	0.64
	Childhood-onset ITP	5	329/497	Dominant	1.02	0.74-1.42	0.13	0.89	R	3.41	0.49	0.00	0.81
				Recessive	1.66	0.90-3.06	1.61	0.11	R	7.68	0.11	47.91	**0.05**
				Overdominant	0.66	0.48-0.90	-2.61	**0.01**	R	3.51	0.49	0.00	0.70
				Allele comparison	1.27	0.88-1.82	1.29	0.20	R	11.00	0.03	63.62	0.29
	Adult-onset ITP	3	193/328	Dominant	0.95	0.49-1.83	-0.16	0.88	R	4.76	0.09	57.97	0.41
				Recessive	0.89	0.52-1.52	-0.45	0.66	R	1.69	0.43	0.00	0.19
				Overdominant	1.13	0.78-1.65	0.64	0.52	R	1.30	0.53	0.00	0.44
				Allele comparison	0.92	0.59-1.43	-0.38	0.70	R	4.62	0.09	56.75	**0.05**
	European	5	317/582	Dominant	0.96	0.65-1.40	-0.23	0.82	R	5.86	0.21	31.70	0.29
				Recessive	1.13	0.78-1.64	0.66	0.51	R	3.23	0.52	0.00	0.08
				Overdominant	0.93	0.69-1.26	-0.48	0.63	R	4.49	0.34	10.85	0.60
				Allele comparison	1.02	0.80-1.29	0.14	0.89	R	5.20	0.26	23.07	**0.05**
	North African	2	172/170	Dominant	1.22	0.64-2.33	0.61	0.54	R	1.59	0.21	36.91	NA
				Recessive	7.67	0.41-145.12	1.36	0.17	R	4.08	0.04	75.51	NA
				Overdominant	0.50	0.30-0.84	-2.60	**0.01**	R	0.57	0.47	0.00	NA
				Allele comparison	1.83	0.72-4.63	1.27	0.21	R	6.42	0.01	84.41	NA

Supplementary Table 2: Meta Analysis of the *FCGR2A* rs1801274 in SLE

Disease	Population	No. of studies	Sample size (cases/controls)	Comparison	Test of Association					Test of Heterogeneity			Publication bias
					OR	95% CI	Z	*P* Value	Model	Q	*P* Value	I^2 (%)	Egger's test *P* Value
SLE	Overall	14	4524/5199	Dominant	1.02	0.89-1.17	0.34	0.74	R	21.86	0.06	40.52	0.61
				Recessive	1.19	0.98-1.44	1.73	0.08	R	33.90	0.00	61.65	0.07
				Overdominant	0.96	0.89-1.05	-0.90	0.37	R	8.64	0.69	0.00	0.10
				Allele comparison	1.08	0.96-1.21	1.21	0.23	R	39.78	0.00	67.32	0.31
	European	4	868/1543	Dominant	1.02	0.84-1.25	0.22	0.82	R	2.40	0.49	0.00	0.32
				Recessive	1.10	0.87-1.41	0.79	0.43	R	3.98	0.27	24.54	0.36
				Overdominant	0.96	0.80-1.14	-0.48	0.63	R	0.54	0.91	0.00	0.61
				Allele comparison	1.06	0.90-1.24	0.66	0.51	R	4.06	0.20	33.43	0.23
	East Asian	3	1408/1228	Dominant	1.18	1.00-1.38	2.01	**0.04**	R	2.05	0.37	2.40	0.17
				Recessive	1.39	1.07-1.80	2.44	**0.02**	R	1.79	0.40	0.00	0.22
				Overdominant	1.05	0.90-1.22	0.56	0.58	R	1.13	0.57	0.00	0.51
				Allele comparison	1.16	1.01-1.34	2.03	**0.04**	R	2.81	0.25	28.82	0.11
	North African	2	227/190	Dominant	0.89	0.57-1.38	-0.54	0.59	R	0.84	0.35	0.00	NA
				Recessive	1.13	0.67-1.89	0.45	0.65	R	1.43	0.23	30.27	NA
				Overdominant	0.83	0.56-1.23	-0.92	0.36	R	0.03	0.86	0.00	NA
				Allele comparison	1.00	0.71-1.43	0.02	0.98	R	1.66	0.19	39.70	NA

Supplementary Table 3: Meta Analysis of the *FCGR2A* rs1801274 in RA

Disease	Population	No. of studies	Sample size (cases/controls)	Comparison	Test of Association					Test of Heterogeneity			Publication bias
					OR	95% CI	Z	*P* Value	Model	Q	*P* Value	I^2 (%)	Egger's test *P* Value
RA	Overall	6	1058/1251	Dominant	0.83	0.69-1.00	-1.97	**0.05**	R	5.39	0.37	7.28	0.96
				Recessive	0.79	0.62-1.01	-1.88	0.06	R	4.88	0.42	0.00	0.93
				Overdominant	0.95	0.79-1.14	-0.60	0.55	R	6.06	0.29	17.54	0.27
				Allele comparison	0.86	0.76-0.97	-2.40	**0.02**	R	4.99	0.43	0.00	0.57
	European	2	328/399	Dominant	0.73	0.50-1.07	-1.63	0.10	R	1.26	0.25	20.65	NA
				Recessive	0.71	0.50-1.00	-1.94	**0.05**	R	0.72	0.39	0.00	NA
				Overdominant	0.99	0.74-1.33	-0.06	0.96	R	0.30	0.59	0.00	NA
				Allele comparison	0.78	0.61-1.01	-1.91	**0.06**	R	1.42	0.22	29.75	NA
	East Asian	3	608/724	Dominant	0.91	0.71-1.17	-0.71	0.48	R	2.43	0.34	17.52	0.90
				Recessive	0.71	0.46-1.09	-1.58	0.12	R	0.55	0.77	0.00	0.99
				Overdominant	1.01	0.79-1.29	0.06	0.95	R	2.40	0.30	16.56	0.75
				Allele comparison	0.90	0.76-1.07	-1.23	0.22	R	1.99	0.36	0.00	0.76

Supplementary Table 4: Meta Analysis of the *FCGR2A* rs1801274 in Guillain-Barré Syndrome and Celiac Disease

Disease	Population	No. of studies	Sample size (cases/controls)	Comparison	Test of Association					Test of Heterogeneity			Publication bias
					OR	95% CI	Z	*P* Value	Model	Q	*P* Value	I^2 (%)	Egger's test *P* Value
Guillain-Barré Syndrome (GBS)	Overall	2	443/664	Dominant	1.06	0.81-1.40	0.42	0.68	R	0.09	0.77	0.00	NA
				Recessive	1.16	0.82-1.66	0.86	0.39	R	1.35	0.24	25.83	NA
				Overdominant	0.95	0.83-1.23	-0.40	0.69	R	1.06	0.29	5.31	NA
				Allele comparison	1.08	0.91-1.29	0.87	0.38	R	0.31	0.62	0.00	NA
Celiac Disease	Overall	2	409/648	Dominant	1.19	0.92-1.55	1.30	0.19	R	0.52	0.48	0.00	NA
				Recessive	1.21	0.87-1.67	1.11	0.27	R	0.51	0.48	0.00	NA
				Overdominant	1.06	0.83-1.35	0.44	0.66	R	0.06	0.78	0.00	NA
				Allele comparison	1.14	0.95-1.37	1.45	0.15	R	0.78	0.38	0.00	NA

Supplementary Table 5: Meta Analysis of the *FCGR3A* rs396991 in ITP

Disease	Population /ITP group	No. of studies	Sample size (cases/controls)	Comparison	Test of Association					Test of Heterogeneity			Publication bias
					OR	95% CI	Z	*P* Value	Model	Q	*P* Value	I^2 (%)	Egger's test *P* Value
ITP	Overall	10	675/965	Dominant	2.67	1.94-3.67	6.05	**< 0.001**	R	16.98	0.05	46.99	0.18
				Recessive	2.38	1.78-3.19	5.83	**< 0.001**	R	4.74	0.86	0.00	0.94
				Overdominant	1.58	1.15-2.17	2.81	**0.005**	R	19.81	0.02	54.57	0.75
				Allele comparison	1.97	1.70-2.29	8.93	**< 0.001**	R	7.37	0.60	0.00	0.62
	Childhood-onset ITP	6	382/539	Dominant	3.47	2.40-5.02	6.60	**< 0.001**	R	6.80	0.24	26.48	0.60
				Recessive	2.57	1.71-3.86	4.53	**< 0.001**	R	2.34	0.80	0.00	0.31
				Overdominant	1.97	1.24-3.12	2.89	**< 0.001**	R	12.69	0.03	60.60	0.94
				Allele comparison	2.19	1.79-2.66	7.74	**< 0.001**	R	2.36	0.79	0.00	0.50
	Adult-onset ITP	4	413/486	Dominant	1.86	1.34-2.57	3.73	**< 0.001**	R	3.01	0.40	0.35	0.65
				Recessive	2.20	1.45-3.35	3.70	**< 0.001**	R	2.14	0.55	0.00	0.20
				Overdominant	1.17	0.86-1.60	1.00	0.32	R	1.25	0.75	0.00	0.84
				Allele comparison	1.72	1.37-2.16	4.72	**< 0.001**	R	2.58	0.46	0.00	0.56
	European	6	371/623	Dominant	2.21	1.56-3.13	4.47	**< 0.001**	R	6.67	0.25	25.01	0.16
				Recessive	2.37	1.63-3.44	4.55	**< 0.001**	R	3.53	0.61	0.00	0.85
				Overdominant	1.33	0.97-1.83	1.75	0.08	R	6.45	0.27	22.42	0.20
				Allele comparison	1.81	1.49-2.20	6.02	**< 0.001**	R	4.58	0.47	0.00	0.67
	North African	2	172/169	Dominant	5.12	1.85-14.19	3.14	**< 0.001**	R	3.75	0.05	73.35	NA
				Recessive	1.81	0.90-3.64	1.67	0.10	R	0.03	0.87	0.00	NA
				Overdominant	3.08	1.97-4.80	4.96	7.11	R	0.99	0.32	0.00	NA
				Allele comparison	2.31	1.68-3.18	5.14	**< 0.001**	R	0.90	0.35	0.00	NA

Supplementary Table 6: Meta Analysis of the *FCGR3A* rs396991 in SLE

Disease	Population	No. of studies	Sample size (cases/controls)	Comparison	Test of Association					Test of Heterogeneity			Publication bias
					OR	95% CI	Z	*P* Value	Model	Q	*P* Value	I^2 (%)	Egger's test *P* Value
SLE	Overall	10	3404/4190	Dominant	0.98	0.77-1.25	-0.15	0.88	R	50.82	0.00	82.29	**0.01**
				Recessive	1.12	0.70-1.80	0.47	0.64	R	72.43	0.00	87.58	0.63
				Overdominant	0.82	0.70-0.97	-2.40	**0.02**	R	20.43	0.02	55.95	0.21
				Allele comparison	1.08	0.83-1.41	0.56	0.58	R	112.18	0.00	91.98	**0.05**
	European	3	659/621	Dominant	0.74	0.57-0.96	-2.31	**0.02**	R	2.30	0.31	12.93	0.19
				Recessive	0.63	0.40-1.00	-1.96	**0.05**	R	1.44	0.50	0.00	0.55
				Overdominant	0.83	0.65-1.06	-1.50	0.13	R	1.75	0.44	0.00	0.21
				Allele comparison	0.77	0.62-0.95	-2.48	**0.01**	R	2.54	0.29	21.13	0.46
	East Asian	2	895/1015	Dominant	0.71	0.59-0.85	-3.80	**< 0.01**	R	0.03	0.83	0.00	NA
				Recessive	0.69	0.49-0.96	-3.62	**0.03**	R	0.55	0.47	0.00	NA
				Overdominant	0.79	0.66-0.95	-2.50	**0.01**	R	0.18	0.66	0.00	NA
				Allele comparison	0.77	0.67-0.88	-3.80	**< 0.01**	R	0.24	0.63	0.00	NA

Supplementary Table 7: Meta Analysis of the *FCGR3A* rs396991 in RA

Disease	Population	No. of studies	Sample size (cases/controls)	Comparison	Test of Association					Test of Heterogeneity			Publication bias
					OR	95% CI	Z	*P* Value	Model	Q	*P* Value	I^2 (%)	Egger's test *P* Value
RA	Overall	6	1772/1556	Dominant	1.14	0.97-1.34	1.57	0.12	R	5.88	0.33	14.92	0.21
				Recessive	1.36	1.09-1.69	2.73	**0.01**	R	1.90	0.86	0.00	0.66
				Overdominant	0.99	0.86-1.15	-0.10	0.92	R	3.52	0.62	0.00	0.30
				Allele comparison	1.15	1.03-1.29	2.44	**0.02**	R	5.49	0.36	8.84	0.35
	European	3	1276/891	Dominant	1.19	0.96-1.48	1.57	0.12	R	2.52	0.30	20.49	0.13
				Recessive	1.41	1.08-1.85	2.51	**0.01**	R	1.36	0.51	0.00	0.26
				Overdominant	1.00	0.84-1.20	0.01	0.99	R	0.92	0.63	0.00	0.39
				Allele comparison	1.21	1.02-1.42	2.21	**0.03**	R	2.74	0.25	27.04	0.11
	East Asian	2	370/536	Dominant	0.96	0.73-1.25	-0.31	0.76	R	0.24	0.62	0.00	NA
				Recessive	1.23	0.81-1.85	0.97	0.33	R	0.21	0.64	0.00	NA
				Overdominant	0.88	0.67-1.15	-0.93	0.35	R	0.01	0.91	0.00	NA
				Allele comparison	1.02	0.84-1.25	0.23	0.82	R	0.50	0.50	0.00	NA

Supplementary Table 8: Meta Analysis of the *FCGR3A* rs396991 in Guillain-Barré Syndrome and Celiac Disease

Disease	Population	No. of studies	Sample size (cases/controls)	Comparison	Test of Association					Test of Heterogeneity			Publication bias
					OR	95% CI	Z	*P* Value	Model	Q	*P* Value	I^2 (%)	Egger's test *P* Value
Guillain-Barré Syndrome (GBS)	Overall	2	443/665	Dominant	0.96	0.75-1.23	-0.33	0.74	R	0.82	0.35	0.00	NA
				Recessive	0.97	0.67-1.42	-0.14	0.89	R	0.06	0.81	0.00	NA
				Overdominant	0.98	0.76-1.25	-0.19	0.85	R	0.48	0.49	0.00	NA
				Allele comparison	1.01	0.87-1.17	0.13	0.90	R	1.00	0.71	0.24	NA
Celiac Disease	Overall	2	443/665	Dominant	0.99	0.76-1.29	-0.08	0.94	R	0.01	0.92	0.00	NA
				Recessive	0.99	0.72-1.36	-0.08	0.94	R	0.02	0.88	0.00	NA
				Overdominant	1.00	0.78-1.28	0.00	1.00	R	0.00	1.00	0.00	NA
				Allele comparison	0.99	0.83-1.18	-0.11	0.91	R	0.03	0.90	0.00	NA

Supplementary Table 9: Meta-analysis of all pooled studies of *FCGR2A* rs1801274

Study	OR	95%	Weight	t	*P*-value
Zakaria 2021 (ITP)	2.86	1.78 - 4.58	0		
Pavkovic 2018 (ITP)	1.16	0.80 - 1.69	0		
Audia 2017 (ITP)	0.50	0.25 - 0.98	0		
Amorim 2012 (ITP)	0.90	0.50 - 1.61	0		
Eyada 2012 (ITP)	1.33	0.81 - 2.19	0		
Breunis 2008 (ITP)	1.07	0.65 - 1.77	0		
Breunis 2008 (ITP)	1.00	0.65 - 1.53	0		
Bruin 2004 (ITP)	1.20	0.77 - 1.87	0		
Cornwell 2023 (SLE)	0.97	0.45 - 2.09	0		
Dhaouadi 2019 (SLE)	0.85	0.59 - 1.22	0		
Tsang 2016 (SLE)	1.00	0.82 - 1.21	0.33		
Vigato-Ferreira 2016 (SLE)	1.44	1.05 - 1.98	0		
Kwon 2016 (SLE)	1.26	1.06 - 1.50	1.21		
Zidan 2013 (SLE)	1.22	0.81 - 1.85	0		
Zhou 2011 (SLE)	1.17	0.98 - 1.40	0.9		
Sánchez 2011 (SLE)	0.84	0.76 - 0.92	97.44		
Jönsen 2007 (SLE)	0.88	0.68 - 1.13	0.01		
Jönsen 2004 (SLE)	1.28	0.94 - 1.74	0		
Magnusson 2004 (SLE)	1.21	0.89 - 1.64	0		
Magnusson 2004 (SLE)	1.00	0.76 - 1.32	0		
Brazilio 2004 (SLE)	1.53	0.95 - 2.47	0		
Chu 2004 (SLE)	0.91	0.65 - 1.28	0		
Sun 2017 (RA)	1.04	0.75 - 1.44	0		
Meziani 2012 (RA)	0.74	0.52 - 1.04	0		
Meziani 2012 (RA)	0.71	0.54 - 0.92	0		
Chen 2006 (RA)	0.89	0.69 - 1.15	0.01		
Morgan 2006 (RA)	0.91	0.65 - 1.28	0		
Morgan 2006 (RA)	0.96	0.67 - 1.36	0		
Hayat 2020 (GBS)	1.12	0.89 - 1.42	0.03		
Dourado 2016 (GBS)	1.03	0.78 - 1.35	0		
Sareneva 2009 (CD)	1.21	0.97 - 1.51	0.06		
Sareneva 2009 (CD)	1.02	0.75 - 1.39	0		
**Random effects model**	**1.05**	**0.96 - 1.14**	**99.99**	**1.1**	**0.27**

ORs and 95% CIs for the outcomes of the allelic comparison in the overall population ($P = 0.27$, OR = 1.05, 95% CI = 0.96-1.14), North African ($P <$ **0.01**, OR = 1.39, 95% CI = 0.84-2.29), and East Asian ($P =$ **0.04**, OR = 1.02, 95% CI = 0.87-1.20). $\tau^2 = 0.03$, 95% CI = 0.012-0.084; $\tau = 0.17$, 95% CI = 0.109-0.290; $I^2 = 0.61$, 95% CI = 0.422-0.733; H = 1.60, 95% CI = 1.316-1.934; Q = 78.87, d.f = 31, *P*-value = 0.

Supplementary Table 10: Meta-analysis of all pooled studies of *FCGR3A* rs396991

Study	OR	95%	Weight	t	*P*-value
Zakaria 2021 (ITP)	2.71	1.71 - 4.29	1.07		
Pavkovic 2018 (ITP)	1.49	1.04 - 2.14	2.26		
Audia 2017 (ITP)	2.27	1.20 - 4.29	0.31		
Papagianni 2013 (ITP)	1.57	0.88 - 2.81	0.45		
Amorim 2012 (ITP)	2.24	1.23 - 4.08	0.39		
Nourse 2012 (ITP)	2.07	1.37 - 3.12	1.54		
Eyada 2012 (ITP)	1.99	1.28 - 3.10	1.22		
Breunis 2008 (ITP)	1.43	0.84 - 2.42	0.64		
Breunis 2008 (ITP)	2.38	1.52 - 3.72	1.17		
Bruin 2004 (ITP)	2.15	1.37 - 3.36	1.16		
Karimifar 2021 (SLE)	5.48	3.67 - 8.19	1.66		
Dhaouadi 2019 (SLE)	2.28	1.57 - 3.32	2.04		
Dong 2014 (SLE)	0.93	0.81 - 1.06	9.59		
Dong 2014 (SLE)	0.98	0.84 - 1.14	8.84		
Dai 2013 (SLE)	0.78	0.67 - 0.90	9.06		
Jönsen 2007 (SLE)	0.65	0.49 - 0.86	4.06		
Jönsen 2004 (SLE)	0.92	0.66 - 1.27	2.91		
Magnusson 2004 (SLE)	0.79	0.54 - 1.15	1.99		
Magnusson 2004 (SLE)	0.90	0.66 - 1.23	3.28		
Chu 2004 (SLE)	0.71	0.50 - 1.00	2.52		
Sun 2017 (RA)	0.93	0.67 - 1.30	2.82		
Thabet 2009 (RA)	1.08	0.91 - 1.28	8.09		
Chen 2006 (RA)	1.08	0.84 - 1.38	5.1		
Morgan 2006 (RA)	1.4	0.99 - 1.98	2.52		
Morgan 2006 (RA)	1.35	0.93 - 1.97	2.03		
Kastbom 2005 (RA)	1.33	1.02 - 1.74	4.49		
Hayat 2020 (GBS)	0.92	0.73 - 1.16	5.68		
Dourado 2016 (GBS)	1.07	0.79 - 1.44	3.55		
Sareneva 2009 (CD)	0.98	0.79 - 1.22	6.21		
Sareneva 2009 (CD)	1.01	0.74 - 1.37	3.36		
**Random effects model**	**1.29**	**1.116 - 1.48**	**100.01**	**3.48**	**< 0.01**

ORs and 95% CIs for the outcomes of the allelic comparison in the overall population ($P <$ **0.01**, OR = 1.29, 95% CI = 1.12-1.48), European ($P <$ **0.01**, OR = 1.23, 95% CI = 1.03-1.47). $\tau^2 = 0.12$, 95% CI = 0.097-0.346; $\tau = 0.35$, 95% CI = 0.312-0.588; $I^2 = 0.86$, 95% CI = 0.810-0.896; H = 2.67, 95% CI = 2.296-3.103; Q = 206.66, d.f = 29, *P*-value = 0.
